# Targeting *A20* Decreases Glioma Stem Cell Survival and Tumor Growth

**DOI:** 10.1371/journal.pbio.1000319

**Published:** 2010-02-23

**Authors:** Anita B. Hjelmeland, Qiulian Wu, Sarah Wickman, Christine Eyler, John Heddleston, Qing Shi, Justin D. Lathia, Jennifer MacSwords, Jeongwu Lee, Roger E. McLendon, Jeremy N. Rich

**Affiliations:** 1 Department of Stem Cell Biology and Regenerative Medicine, Lerner Research Institute, Cleveland Clinic, Cleveland, Ohio, United States of America; 2 Department of Surgery, Duke University Medical Center, Durham, North Carolina, United States of America; 3 Department of Pathology, Duke University Medical Center, Durham, North Carolina, United States of America; University of Rochester Medical Center, United States of America

## Abstract

Glioblastomas are deadly cancers that display a functional cellular hierarchy maintained by self-renewing glioblastoma stem cells (GSCs). GSCs are regulated by molecular pathways distinct from the bulk tumor that may be useful therapeutic targets. We determined that A20 (TNFAIP3), a regulator of cell survival and the NF-κB pathway, is overexpressed in GSCs relative to non-stem glioblastoma cells at both the mRNA and protein levels. To determine the functional significance of A20 in GSCs, we targeted *A20* expression with lentiviral-mediated delivery of short hairpin RNA (shRNA). Inhibiting A20 expression decreased GSC growth and survival through mechanisms associated with decreased cell-cycle progression and decreased phosphorylation of p65/RelA. Elevated levels of A20 in GSCs contributed to apoptotic resistance: GSCs were less susceptible to TNFα-induced cell death than matched non-stem glioma cells, but *A20* knockdown sensitized GSCs to TNFα-mediated apoptosis. The decreased survival of GSCs upon *A20* knockdown contributed to the reduced ability of these cells to self-renew in primary and secondary neurosphere formation assays. The tumorigenic potential of GSCs was decreased with *A20* targeting, resulting in increased survival of mice bearing human glioma xenografts. In silico analysis of a glioma patient genomic database indicates that A20 overexpression and amplification is inversely correlated with survival. Together these data indicate that A20 contributes to glioma maintenance through effects on the glioma stem cell subpopulation. Although inactivating mutations in *A20* in lymphoma suggest A20 can act as a tumor suppressor, similar point mutations have not been identified through glioma genomic sequencing: in fact, our data suggest A20 may function as a tumor enhancer in glioma through promotion of GSC survival. A20 anticancer therapies should therefore be viewed with caution as effects will likely differ depending on the tumor type.

## Abstract 2

The A20 protein is a known inhibitor of apoptosis that here is shown to be a novel cancer stem cell-promoting factor associated with poor glioma patient survival.

## Author Summary

Glioblastomas are the most common and aggressive primary brain tumors in adults, with a median survival of only 12–15 months. Glioblastomas display a cellular hierarchy with a subset of cells having stem cell–like properties, including the capacity to self-renew and propagate tumors. Specific ablation of cancer stem cells is widely thought to be critical for effective and long-lasting treatment of cancers. We report the identification of the antiapoptotic protein A20 (which is also known as TNFAIP3) as a novel regulator of glioma stem cell survival. Glioma stem cells overexpress A20 relative to non-stem glioma cells, and this protects them from cell death, whereas depletion of A20 attenuates glioma stem cell survival and tumor growth. Interrogation of a molecular glioma database reveals that A20 levels correlate with decreased survival in patients. These data indicate that A20 is a tumor enhancer in the context of glioma, which importantly contrasts with its known function as a tumor suppressor in the context of lymphoma. Therefore, A20 may be a context-specific regulator of cancer stem cell survival and growth.

## Introduction

Tumors are aberrant organ systems that display a complex interplay between neoplastic cells and recruited vascular, inflammatory, and stromal elements [1]. Cellular heterogeneity within the neoplastic compartment has been modeled with complementary stochastic and hierarchical paradigms. Molecular signals that drive tumor formation and maintenance frequently are shared with normal development and wound responses, processes in which normal stem and progenitor cells function [1]–[4]. Stem cell–like cancer cells (or cancer stem cells) need not be derived from normal stem cells but may be subjected to evolutionary pressures that select for the capacity to self-renew extensively or differentiate depending on conditions [1]–[5]. Cancer stem cells have been derived from several primary brain tumors, but both their derivation and characterization are incomplete and rapidly expanding [6]–[35]. Glioblastoma (World Heath Organization grade IV astrocytoma) is the most common primary brain tumor in adults and one of the most aggressive and deadly cancers [36],[37]. Current glioblastoma therapies, including radiotherapy and chemotherapy, are highly toxic, offering only palliation [36],[37]. Although brain tumor stem cells remain controversial due to the evolving understanding of their nature, a number of reports have demonstrated that glioblastomas contain cancer stem cells and that these cells contribute to therapeutic resistance and tumor angiogenesis [1]–[35]. Significant effort has been undertaken to identify potential targets in cancer stem cells that promote tumor maintenance and that might be amenable to disruption [35].

To identify molecular targets in cancer, the majority of analyses completed to date compare bulk tumor to normal tissues and may therefore underestimate the importance of genes and proteins expressed within the cancer stem cell subpopulation. For example, comparison of GSCs to non-stem glioma cells or bulk tumor has resulted in greater understanding of the importance of HIF2α [10], L1CAM [21], and Bmi-1 [22] for GSC tumorigenic capacity. These proteins are all relatively overexpressed in glioblastoma stem cells (GSCs) and now known to regulate GSC growth, survival, and self-renewal [10],[21],[22]. These biological processes are also regulated in GSCs by more well-established molecular mediators of cancer such as c-myc [23] and AKT [24]–[26]. Together, these studies demonstrate that isolation and characterization of GSCs can define new molecular targets for cancer therapy and determine novel roles for established signaling pathways in cancer stem cell biology.

We speculated that the cell survival and NF-κB regulator A20, or Tumor Necrosis Factor α inducible protein 3 (TNFAIP3), was one molecular target with a greater role in glioma than currently understood [38],[39]. An oncogenic role for A20 is suggested by the increased *A20* expression in some cancers: A20 is elevated in undifferentiated nasopharyngeal carcinoma [40], poorly differentiated head and neck cancers [40], gliomas [41], and inflammatory breast cancer [42]. Increased A20 expression in breast cancer cells confers resistance to TNFα [43],[44] and tamoxifen [45], suggesting A20 mediates survival and chemoresistance. Although these data imply a protumorigenic role for A20, gene expression and functional studies in other cancer types suggest A20 is a tumor suppressor. Deletions and inactivating mutations have been identified in B-cell lymphoma, Hodgkin lymphomas, and non-Hodgkin lymphomas [46]–[51]. In addition, decreased A20 was associated with resistance to DNA-damaging agents in glioma cells [52]. Together, these data indicate that the role of A20 in cancer biology may be context and tissue-type dependent and is an important area for further investigation. Evidence from the *A20* null mice also suggested a link to stem cell biology: the epidermal and dermal layers of *A20^−/−^* mice are significantly thicker than that of wild-type controls, demonstrating A20 regulates skin cell fate [53]. Based on these data, we examined the role of A20 in cancer stem cell biology in gliomas. We determined A20 is elevated in GSCs where it is required to maintain growth and survival. Importantly, we find increased *A20* mRNA expression or copy number is associated with poor glioma patient survival.

## Results

### A20 Is Highly Expressed in Glioma Stem Cells

**Figure 1:**
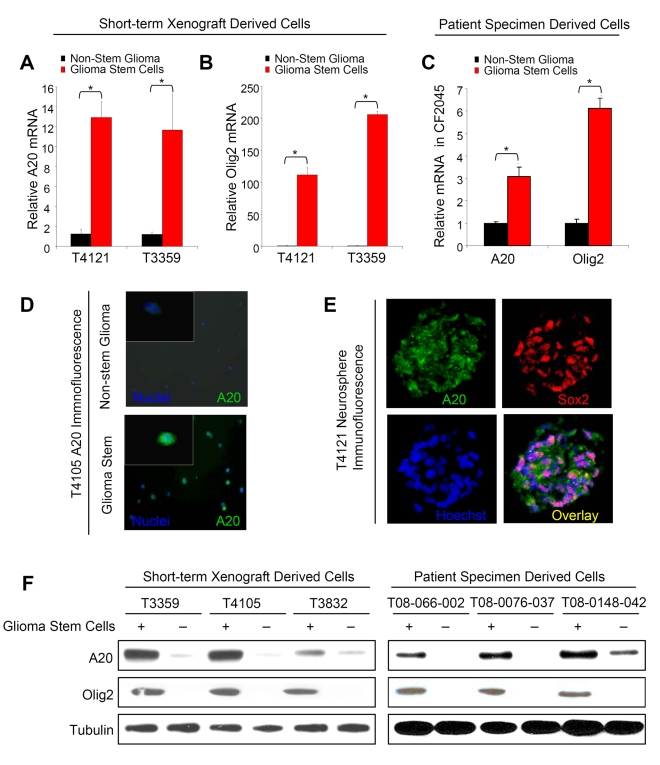
A20 expression is elevated in glioma stem cells. (A) *A20* mRNA expression is elevated in GSCs isolated from T4121 and T3359 glioma patient specimens passaged short term in immunocompromised mice in comparison to matched non-stem glioma cells. An asterisk (*) indicates *p*<0.05 with *t*-test comparison of glioma stem cells and non-stem glioma cells isolated from the same tumor. (B) Isolated glioma stem cells utilized in (A) express increased mRNA levels of the GSC marker Olig2 in comparison to matched non-stem glioma cells. An asterisk (*) indicates *p*<0.01 with *t*-test comparison of glioma stem cells and non-stem glioma cells isolated from the same tumor. (C) *A20* mRNA expression is elevated in GSCs isolated from a CCF2045 patient specimen passaged in comparison to matched non-stem glioma cells. An asterisk (*) indicates *p*<0.05 with *t*-test comparison of glioma stem cells and non-stem glioma cells isolated from the same tumor. (D) Immunofluorescent staining of isolated glioma stem cells from a T4105 glioma patient specimen passaged short term in immunocompromised mice demonstrates increased A20 protein expression in comparison to matched non-stem glioma cells. (E) Immunofluorescent staining demonstrates A20 expression in neurospheres that also express the stem cell transcription factor Sox2. (F) Western blot analysis demonstrates glioma stem cells isolated from glioma specimens passaged short term in immunocompromised mice (T3359, T4105, T3832) or directly from patient specimens (T066002, T0076037, T0148042) express more A20 than matched non-stem glioma cells. Expression of the glioma stem cell marker Olig2 is elevated in isolated glioma stem cells. Tubulin expression demonstrates equivalent protein loading.

To determine whether A20 could differentially contribute to glioma biology through the recently identified glioma subfractions, we utilized several complementary methods to evaluate A20 expression in freshly isolated GSC-enriched and -depleted cultures derived using our previously published methodology [11],[12],[15],[21],[23],[25]. To determine whether A20 was differentially expressed at the transcript level, total mRNA was collected and analyzed by quantitative real-time PCR. We found GSC-enriched cells isolated from short-term xenografts expressed elevated *A20* mRNA levels in comparison to matched non-stem glioma cells (Figure 1A). We had previously confirmed that these cells self-renew and propagate tumors in an immunocompromised host [11],[12],[21],[23]. As in our past studies, the GSCs also express high levels of the glioma stem cell marker encoded by *Olig2* (Figure 1B). Consistent with these results, GSCs isolated directly from patient specimens also expressed elevated levels of A20 and Olig2 (Figure 1C). To determine whether elevated *A20* mRNA expression correlated with increased A20 protein levels, we next visualized A20 expression using immunofluorescence (Figure 1D and 1E). Increased expression of A20 was observed in GSCs in comparison to matched non-stem glioma cells (Figure 1D). GSCs form neurospheres when cultured in serum-free media. When single neurospheres were sectioned, we determined A20 was coexpressed with the stem cell transcription factor Sox2 (Figure 1E). To confirm these results, we enriched or depleted GSCs from either short-term xenografts or patient tumor specimens and isolated lysates for immunoblotting (Figure 1F; Figure S1). In every tumor tested, GSCs displayed strikingly elevated A20 levels compared to matched non-stem cells. Similar to results with mRNA, the differential expression of A20 was true whether subfractions were isolated from patient specimens passaged short term in immunocompromised mice or directly from patient specimens (Figure 1F; Figure S1).

**Figure 2:**
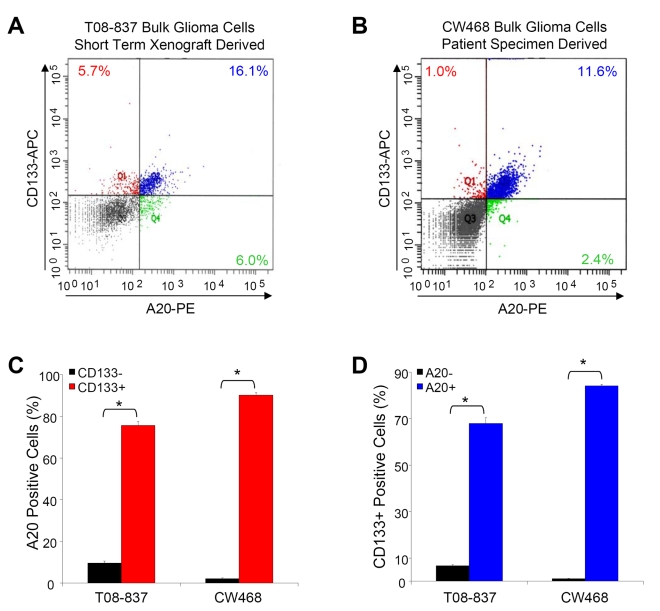
Flow cytometry demonstrates A20 colocalizes with a glioma stem cell marker. (A and B) Flow cytometry of fixed bulk tumor cells isolated from T08-837 short-term xenograft or directly from a CW468 patient specimen demonstrates significant co-staining of the glioma stem cell marker CD133 and A20. Representative FACS plots are shown for T08-837 (A) and CW468 (B). (C) When the percentage of A20+ cells is determined in the CD133− and CD133+ fractions, significantly higher percentages of A20+ cells are present in the CD133+ fraction. (D) When the percentage of CD133+ cells is determined in the A20− and A20+ fractions, significantly higher percentages of CD133+ cells are present in the A20+ fraction. An asterisk (*) indicates *p*<0.001 with *t*-test comparison of the indicated samples.

To further evaluate whether cells expressing a GSC marker also highly express A20 at the single-cell level in a quantitative manner, we performed flow cytometric analysis with cells double labeled for A20 and CD133. We confirmed that A20 is highly coexpressed with the glioma stem cell marker CD133 (Prominin-1) when cells were isolated from a patient specimen passaged short term in immunocompromised mice (Figure 2A) or directly isolated from patient specimens (Figure 2B; Figure S2A and S2B). When bulk tumor cells were analyzed for expression of CD133 and A20, greater than 75% of CD133+ cells were also A20+, whereas less than 10% of CD133− cells were A20+ (Figure 2C; Figure S2A and 2SB). The percentage of CD133+ cells is also higher in the A20+ subpopulation: greater than 50% of A20+ cells were CD133+, whereas less than 8% of A20− cells were CD133+ (Figure 2D; Figure S2C and S2D). Coexpression of CD133 and A20 also occurred when cells were cultured short term in vitro after enrichment or depletion of GSCs from a xenografted patient specimen (Figure S2C and S2D). Together, these data strongly support elevated expression levels of A20 in GSCs.

### Expression of shRNA Directed against *A20* Decreases GSC Growth and Survival

**Figure 3:**
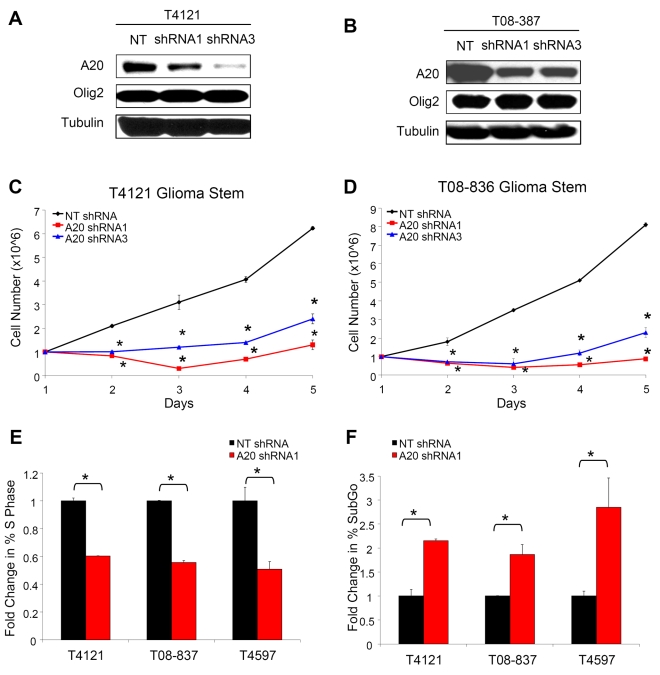
Targeting *A20* decreases glioma stem cell growth. (A and B) Western analysis demonstrates that infection of glioma stem cells isolated from a T4121 (A) orT08-837 (B) patient specimen passaged short term in immunocompromised mice with lentivirus expressing shRNA directed against *A20* decreases A20 protein expression in comparison to infection with lentivirus expressing nontargeting (NT) shRNA. Tubulin expression demonstrates equivalent protein loading. (C and D) Cell counts with Trypan Blue staining demonstrates that decreasing expression of A20 reduces the growth of glioma stem cells isolated from T4121 (C) or T08-836 (D) patient specimens passaged short term in immunocompromised mice. An asterisk (*) indicates *p*<0.05 with ANOVA comparison of nontargeting shRNA to *A20* shRNAs. (E) Cell-cycle analysis demonstrates that targeting *A20* expression decreases the percentage of cells in the S phase of the cell cycle in glioma stem cells isolated from T4121, T08-837, and T4597 cells patient specimen passaged short term in immunocompromised mice. An asterisk (*) indicates *p*<0.05 with *t*-test comparison of nontargeting shRNA control and *A20* shRNA. (F) Cell-cycle analysis demonstrates that targeting A20 expression increases the percentage of cells in the SubGo phase of the cell cycle in isolated T4121, T08-837, and T4597 GSCs. An asterisk (*) indicates *p*<0.05 with *t*-test comparison of nontargeting shRNA control and *A20* shRNA.

Although our findings demonstrated A20 was consistently up-regulated in GSCs, no studies to date have suggested a functional role for A20 in cancer stem cells. As A20 was linked to cell survival in some reports [53]–[57], we first assessed the ability of A20 to regulate GSC cell growth and apoptosis by targeting *A20* expression using lentiviral transduced short hairpin RNAs (shRNAs) (Sigma Mission shRNA). To control for potential off-target shRNA effects, two different sequences of shRNA directed against *A20* and a nontargeting shRNA were used when cell numbers permitted. Transduction with *A20* shRNA reduced A20 protein levels in GSCs in comparison to the nontargeting control, but did not alter Olig2 expression (Figure 3A and 3B). *A20* targeting profoundly impacted GSC growth as demonstrated by a marked reduction in cell numbers over time (Figure 3C and 3D). In contrast, *A20* knockdown minimally altered the growth patterns of non-stem glioma cells (Figure S3A and S3B). The differential dependence of A20 in GSCs and non-stem cells can be further demonstrated by analysis of the relative effect of *A20* shRNA on cell growth (Figure S3C–S3F). To determine whether the decreased growth of GSCs with *A20* knockdown was associated with changes in the cell cycle, we performed flow cytometric analysis with DNA content determination. Cellular entry into S phase was decreased with *A20* targeting (Figure 3E), supporting a role for A20 in proliferation. We also observed an increase in the percentage of cells in the SubG0 phase of the cell cycle (Figure 3F) and a consistent, but relatively modest, 1.1–1.3-fold increase in G_1_ phase cell cycle arrest (unpublished data). Thus, knockdown of *A20* inhibits cell growth due in part to decreased proliferation associated with increased cell death and cell-cycle arrest.

**Figure 4:**
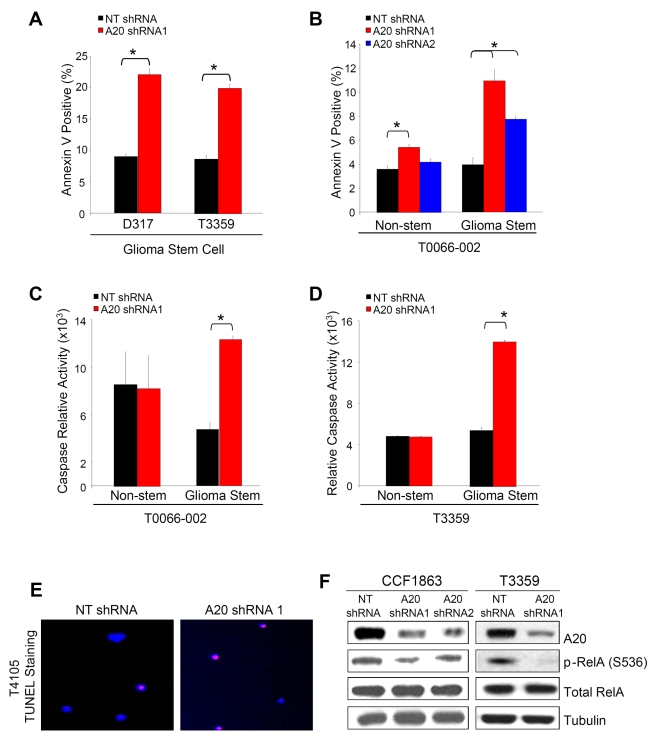
Targeting *A20* decreases glioma stem cell survival. (A) Annexin V staining demonstrates increased apoptosis with *A20* targeting in glioma stem cells isolated from a D317 glioma xenograft or a T3359 patient specimen passaged short term in immunocompromised mice. An asterisk (*) indicates *p*<0.01 with *t*-test comparison of nontargeting (NT) shRNA control and *A20* shRNA. (B) Annexin V staining demonstrates increased apoptosis with *A20* targeting in tumor subfractions isolated from a T0066-002 patient specimen. An asterisk (*), *p*<0.05 with ANOVA comparison of nontargeting shRNA control to the indicated *A20* shRNA. (C) Caspase activity relative to cell number as determined in the cell titer assay increased in *A20* shRNA infected glioma stem cells isolated directly from a T0066-022 patient specimen, but not in matched non-stem glioma cells. An asterisk (*) indicates *p*<0.05 with *t*-test comparison of non-targeting shRNA control to *A20* shRNA. (D) Caspase activity relative to cell number as determined in the cell titer assay increased in *A20* shRNA infected glioma stem cells isolated from a T3359 patient specimen passaged short-term in immunocompromised mice. An asterisk (*) indicates *p*<0.05 with ANOVA comparison of non-targeting shRNA control to the indicated *A20* shRNA. (E) TUNEL staining demonstrates increased apoptosis in A20 infected glioma stem cells isolated from a T4105 patient specimen passaged short term in immunocompromised mice. (F) Targeting *A20* in GSCs isolated from CCF1863 or T3359 patient specimens passaged short term in immunocompromised mice decreased phosphorylation of p65/RelA on Serine 536 as determined via Western blot analysis.

As changes in the cell cycle suggest that A20 regulates cell survival, we evaluated apoptosis with complementary assays. Annexin V assays detect phosphatidylserine expression on the cell surface, a process that occurs during apoptosis and other forms of cell death [5],[58]. In GSCs isolated from two different human glioma xenografts (Figure 4A) and directly from a patient specimen (Figure 4B), introduction of *A20*-directed shRNA increased the percentage of Annexin V–positive cells when compared to nontargeting control shRNA. Caspases, including caspase 3 and caspase 7, are cysteine-aspartic acid proteases that are activated during apoptosis [5],[58]. In GSCs isolated directly from a patient specimen (Figure 4C) or from a human patient specimen passaged short term in immunocompromised mice (Figure 4D), caspase 3/7 activity normalized to cell number increased with *A20* targeting compared to control. Terminal deoxynucleotidyl transferase dUTP nick end labeling (TUNEL) staining detects DNA fragments that occur in the last phase of apoptosis [5],[58]. Increased TUNEL staining was observed in isolated GSCs with knockdown of *A20* (Figure 4E). The potent induction of apoptosis with *A20* targeting was restricted to GSCs as minimal effects were observed with decreased A20 expression in matched non-stem glioma cells (Figure 4B–4D). Together, these data demonstrate that targeting *A20* in GSCs results in increased apoptosis and suggest that A20 is a prosurvival factor for GSCs.

To evaluate the potential mechanism for the decreased survival of GSCs with *A20* targeting, we determined activation of p65/RelA, a central component of NF-κB signaling [59]–[63]. Phosphorylation of RelA at Serine 536 is known to enhance the transactivation potential of the NF-κB complex [62],[63]. NF-κB signals have been shown to be elevated in glioma [64], and targeting RelA in glioma has been shown to decrease cell growth [65]. We found that knockdown of *A20* decreased the activating phosphorylation of RelA without changes in total RelA levels (Figure 4F). These data suggest that failure to maintain active RelA in GSCs contributes, at least in part, to the decreased growth and survival of GSCs with *A20* knockdown.

### Neurosphere Formation Capacity Is Decreased with *A20* Targeting

**Figure 5:**
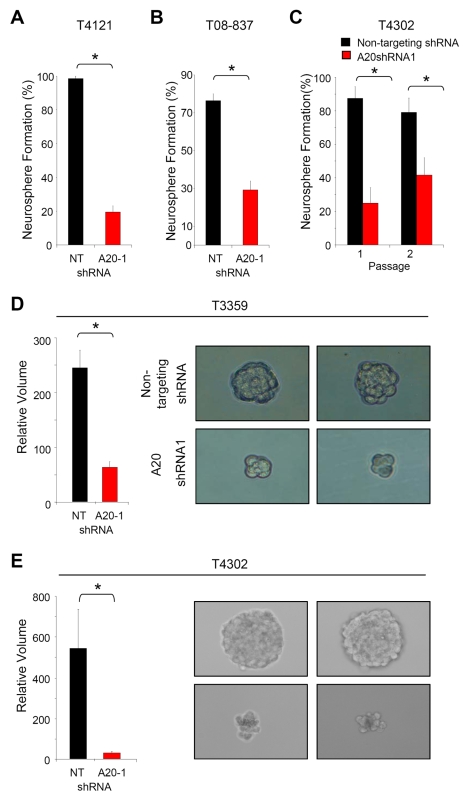
*A20* knockdown decreases neurosphere formation. (A and B) The percentage of wells with neurospheres is decreased in A20-infected glioma stem cells isolated from a T4121 (A) or T08-837 (B) patient specimen passaged short term in immunocompromised mice were infected with *A20* shRNA in comparison to nontargeting (NT) shRNA infected cells. An asterisk (*) indicates *p*<0.01 with *t*-test comparison of nontargeting control shRNA to *A20* shRNA. (C) The percentage of wells with neurospheres is decreased in A20-infected glioma stem cells isolated from a T4302 patient specimen passaged short term in immunocompromised mice in primary and secondary passages. An asterisk (*) indicates *p*<0.05 with *t*-test comparison of nontargeting control shRNA to *A20* shRNA. (D and E) The average volume of spheres formed in neurosphere formation assays with glioma stem cells isolated from a T3359 (D) or T4302 (E) patient specimen passaged short term in immunocompromised mice is decreased with infection of *A20* shRNA in comparison to nontargeting shRNA. Representative images of spheres used for analysis of volume are shown.

Cancer stem cells are functionally defined through their capacity for sustained self-renewal. As the growth and survival of GSCs was affected by *A20* knockdown, we next examined whether A20 was important for self-renewal. To more definitively evaluate this possibility, we utilized an in vitro indicator of self-renewal in normal and cancer stem cells: the neurosphere assay [1]–[9],[66]–[68]. We found that targeting *A20* in GSCs decreased neurospheres formation in comparison to cells transduced with nontargeting control shRNA (Figure 5). A20 loss decreased the percentage of wells with neurospheres in both primary (Figure 5A–5C) and secondary (Figure 5C) passages. Neurospheres that did form from *A20*-targeted GSCs were smaller than those forming from nontargeted GSCs (Figure 5D–5E), suggesting decreased proliferation. Thus, the formation of neurospheres is significantly hampered by the loss of A20, indicating a role for A20 in GSC self-renewal.

### A20 Protects GSCs from TNFα-Induced Apoptosis

**Figure 6:**
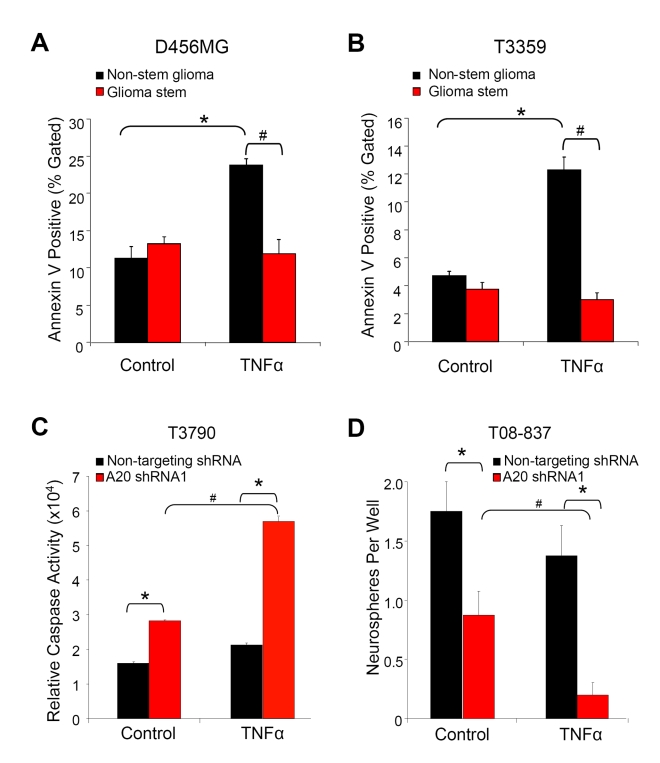
A20 protects GSCs from TNFα-induced apoptosis. (A and B) The percentage of Annexin V–positive cells increases with TNFα treatment of non-stem glioma cells, but not matched GSC-enriched cultures, isolated from a D456MG (A) or a T3359 (B) patient specimen passaged short term in immunocompromised mice. An asterisk (*) indicates *p*<0.01 with ANOVA comparison to untreated non-stem glioma cells. A number sign (#) indicates *p*<0.01 with ANOVA comparison of similarly treated non-stem glioma and glioma stem cells. (C) Targeting *A20* in GSCs sensitizes to TNFα-induced apoptosis. GSCs isolated from a T3790 glioma xenograft infected with nontargeting shRNA or shRNA directed against *A20* were treated with 5 ng/ml TNFα and relative caspase activity measured. An asterisk (*) indicates *p*<0.05 with ANOVA comparison to similarly treated non-targeting control cells. A number sign (#) indicates *p*<0.05 with ANOVA comparison of TNFα to untreated cells infected with the same shRNA. (D) TNFα augments decreases in neurosphere formation observed in GSCs with *A20* targeting. Ten cells per well were plated in the neurosphere formation assay and subsequently treated with 5 ng/ml TNFα. After 12 d, the number of neurospheres per well was assessed. An asterisk (*) indicates *p*<0.05 with ANOVA comparison of nontargeting control shRNA to *A20* shRNA. A number sign (#) indicates *p*<0.05 with ANOVA comparison of control versus TNFα-treated *A20* shRNA-treated GSCs.

According to the cancer stem cell hypothesis, GSCs and other cancer stem cells are responsible for tumor maintenance and recurrence after therapy due to the ability of these cells to survive cellular assaults that would typically result in cell death [1]–[5]. For example, GSCs have been shown to preferentially survive radiotherapy [15],[16], chemotherapy [17],[18], treatment with TNF-related apoptosis-inducing ligand (TRAIL) [19], and Fas-induced apoptosis [20]. These data demonstrate GSCs are resistant to a wide variety of prodeath signals. Prior studies suggested that TNFα therapy may be beneficial for the treatment of glioma due to the ability to promote apoptosis [69], but the sensitivity of cancer stem cells to this effect has not been determined. As A20 can inhibit TNF-induced apoptosis in some cell types [43],[44],[53]–[57], and A20 levels are elevated in GSCs, we hypothesized that GSCs are resistant to TNFα-induced apoptosis. We therefore investigated the effect of TNFα on the survival of GSC-enriched and -depleted cultures. TNFα increased apoptosis in non-stem glioma cells (Figure 6A and 6B), consistent with prior results in glioma cell lines [70],[71]. In contrast, GSCs isolated from multiple human glioma xenografts were resistant to TNFα-induced cell death (Figure 6A and 6B and unpublished data). To determine the role of A20 in GSC TNFα apoptotic resistance, GSCs were transduced with either shRNA directed against *A20* or nontargeting shRNA and subsequently treated with TNFα. Consistent with our prior results (Figure 4), GSC apoptosis was increased with targeting of *A20* as measured with caspase activity (Figure 6C; Figure S4A). This increase in apoptosis was significantly enhanced when *A20* knockdown cells were additionally treated with TNFα (Figure 6C; Figure S4A). To determine whether the TNFα effects could differentially regulate self-renewal in *A20* knockdown cells, we evaluated neurosphere formation in the presence and absence of *A20* targeting and TNFα treatments. We found that addition of TNFα in the presence of *A20* shRNA, but not nontargeting shRNA, significantly decreased neurosphere formation (Figure 6D; Figure S4B). Together, these data indicate that GSCs are resistant to TNFα-induced apoptosis in an A20-dependent manner and further demonstrate A20 is an important prosurvival factor in GSCs.

### Targeting *A20* Increases the Survival of Mice Bearing Human Glioma Xenografts

**Figure 7:**
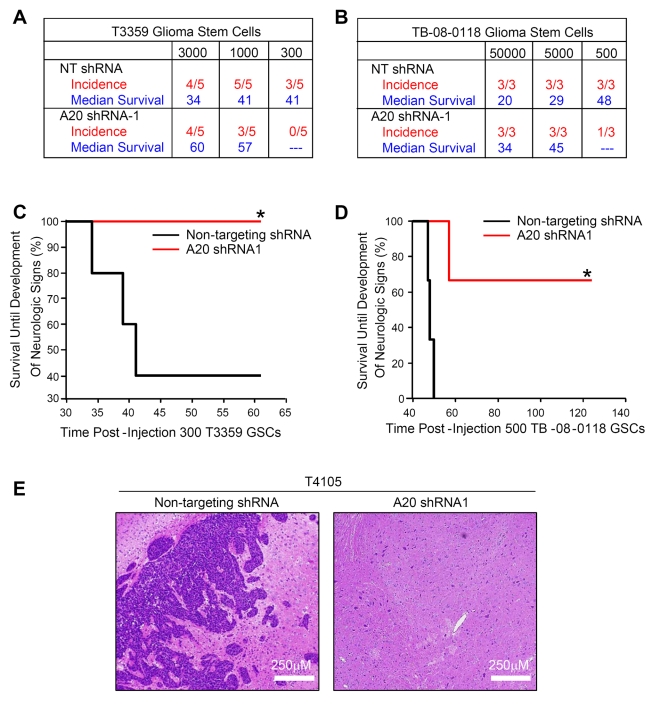
Targeting *A20* decreases glioma stem cell tumorigenic potential and increases the survival of mice bearing intracranial human glioma xenografts. (A) Median survival of mice bearing human glioma xenografts increases when injected with *A20* knockdown glioma stem cells in comparison to nontargeting (NT) cells isolated from a T3359 patient specimen passaged short term in immunocompromised mice in the in vivo limiting dilution assay. (B) Median survival of mice bearing human glioma xenografts increases when injected with *A20* knockdown glioma stem cells in comparison to nontargeting cells isolated from a T080118 patient specimen in the in vivo limiting dilution assay. (C) Kaplan-Meier curves demonstrate increased survival with *A20* targeting in Balbc nu/nu mice injected with 300 glioma stem cells isolated from a T3359 xenograft. An asterisk (*) indicates *p*<0.05 with log-rank analysis of survival curves. (D) Kaplan-Meier curves demonstrate increased survival with *A20* targeting when 500 glioma stem cells isolated from a T080118 glioma patient specimen were injected into Balbc nu/nu mice. An asterisk (*) indicates *p*<0.05 with log-rank analysis of survival curves. (E) Representative images of brains of mice injected with T4105 GSCs infected with lentivirus expressing nontargeting shRNA or shRNA directed against *A20*. For this experiment, all animals were sacrificed upon the development of the first neurologic sign in any one mouse (a nontargeting shRNA animal). Hematoxylin and eosin staining demonstrated the presence of brain tumors in mice injected with nontargeting shRNA infected GSCs, including the presence of glioma cells infiltrating normal brain. Gliomas were not observed in brains of mice injected with *A20* knockdown GSCs.

Our results thus far determined an important role for A20 in GSC growth, survival, and self-renewal in vitro, but the ultimate goal of any cancer stem cell–directed therapy is to provide therapeutic benefit in vivo. We therefore evaluated the ability of *A20* targeting to increase the survival of immunocompromised mice bearing intracranially implanted human glioma cells. For initial experiments, we performed an in vivo limiting dilution assay with GSCs transduced with nontargeting control shRNA or shRNA directed against *A20*. Tumor-bearing mice were allowed to survive until the development of neurologic signs in each animal (including lethargy, ataxia, paralysis, or seizure) in accordance with Institutional Animal Care and Use Committee–approved protocols. For all cell numbers transplanted, the median survival of mice injected with GSCs derived from either T3359 (Figure 7A) or TB-08-0118 (Figure 7B) was increased with *A20* knockdown. When 300–1,000 cells were injected, the tumor incidence also decreased when *A20* was targeted (Figure 7A and 7B). Kaplan-Meier curves further demonstrate significant increases in survival with introduction of *A20* shRNA when T3359 (Figure 7C) or TB-08-0118 (Figure 7D) GSCs were injected. Although cells used in the in vivo studies displayed successful targeting of A20 expression prior to implantation, immunohistochemical analysis of *A20* shRNA tumors that grew out showed that these tumors escaped *A20* knockdown (Figure S5). In a separate experiment with T4105 cells in which all animals were sacrificed simultaneously at the onset of the first neurologic sign in any animal, infiltrating glioma cells were observed in animals injected with GSCs infected with nontargeting shRNA, but not *A20*-targeting shRNA (Figure 7E). These in vivo data indicate that targeting *A20* in GSCs can increase survival in mouse models of human brain tumors and suggest A20 could be a useful therapeutic target in glioma.

### A20 Expression Is Elevated in Human Glioma Patients and Correlates with Poor Survival

**Figure 8:**
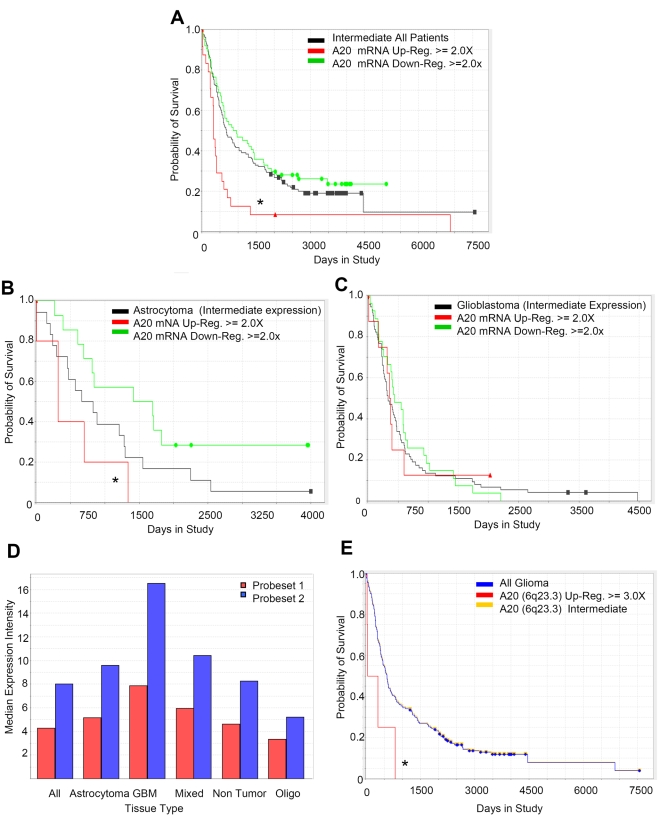
A20 is a prognostic indicator in glioma patients. (A) Increased *A20* mRNA expression was associated with reduced glioma patient survival in REMBRANDT. Twenty-four patients exhibited a 2-fold elevation in mRNA, whereas 64 patients were classified as having down-regulated expression and 130 with intermediate expression. An asterisk (*) indicates *p*<0.006 with log-rank analysis of survival curves for up-regulated versus down-regulated expressing groups. (B) Increased *A20* mRNA expression was associated with reduced astrocytoma patient survival in REMBRANDT. Five patients exhibited a 2-fold elevation in mRNA, whereas 14 patients were classified as having down-regulated expression and 18 with intermediate expression. An asterisk (*) indicates *p*<0.009 with log-rank analysis of survival curves for up-regulated versus down-regulated expressing groups. (C) *A20* mRNA expression was not associated with survival in glioblastoma patients in REMBRANDT. Eight patients exhibited a 2-fold elevation in mRNA, whereas 27 patients were classified as having down-regulated expression and 74 with intermediate expression. *p*<0.68 with log-rank analysis of survival curves for up-regulated versus down-regulated expressing groups. (D) The median intensity of A20 expression is increased in glioblastoma patient samples in REMBRANDT in comparison to all other types of brain tumors when two different probesets are used. (E) A 3-fold change in *A20* (6q23.3) gene amplification was associated with reduced glioma patient survival in REMBRANDT. Four patients exhibited amplification of the *A20* gene compared to 196 intermediate expressing patients. An asterisk (*) indicates *p*<0.0269 with log-rank analysis of survival curves for up-regulated versus intermediate expressing groups.

Our data in human glioma patient specimens and xenografts in vitro and in vivo suggested that elevated A20 levels in GSCs are protumorigenic. To extend these results into a clinical analysis, we utilized two databases: the National Cancer Institute's Repository for Molecular Brain Neoplasia Data (REMBRANDT) and The Cancer Genome Atlas (TCGA), which respectively contain information from multiple brain tumor types or glioblastoma only. Using REMBRANDT, we found that up-regulation of *A20* mRNA 2-fold or greater in all glioma patients correlated with a significant decrease in survival (Figure 8A). When the analysis was restricted to Grade II or Grade III astrocytoma, *A20* mRNA up-regulation 2-fold or greater also significantly correlated with decreased survival (Figure 8B). However, *A20* mRNA up-regulation did not correlate with survival in glioblastoma patients in either REMBRANDT or TCGA (Figure 8C and unpublished data). These data demonstrate that elevation of *A20* in early clinical stages of glioma correlates with poor survival, but differences in *A20* expression in the bulk tumor at the time of glioblastoma diagnosis cannot predict survival. As global gene expression analyses currently available in the public databases cannot consider the contribution of the GSC subfraction, these data may underestimate the importance of A20 in glioblastoma. However, it remained possible that an overall elevation of *A20* in glioblastoma patients compared to lower grade astrocytomas contributed to the poorer survival of glioblastoma patients. To evaluate this possibility, we compared the median expression intensity of *A20* across tumor types in REMBRANDT. We found elevated levels of *A20* in glioblastoma compared to all other types of brain tumors as well as control (nontumor) tissue (Figure 8D). As elevated levels of mRNA may reflect an increase in gene copy number, we sought to determine whether genomic changes in *A20* occurred in glioma patients. Copy number REMBRANDT analysis for *A20* indicated that a 3-fold or greater amplification of the 6q23.3 chromosomal region correlated with poor survival, although the number of patients in this group was small (*n* = 4; Figure 8E). It is also important to note that, in contrast to lymphoma [46]–[51], inactivating point mutations in *A20* were not identified in recently completed genetic screens of the glioma genomes [72],[73]. These data further suggest that A20 activity is important for glioma development and biology. Overall, our data demonstrate that A20 promotes the tumor initiating capacity of GSCs and strongly suggests that increased A20 expression contributes to poor glioma patient outcome.

Understanding the genetic and/or protein expression profile of patients in which A20 is elevated may be important for elucidating the mechanisms through which *A20* is induced or the pathways through which A20-expressing cells regulate survival. We therefore utilized the TCGA glioblastoma database to evaluate the presence of common glioblastoma mutations (Figure S6A and S6B) and expression of TNFα signaling mediators (Figure S6C) in patients with different levels of *A20* expression. Tumors with *TP53* and *NF1* mutations were enriched in tumors with high *A20* expression in comparison to tumors with intermediate or low *A20* levels (Figure S6A and S6B). In contrast, the percentage of samples with *EGFR* mutations was lowest in tumors with high *A20* expression (Figure S6A and S6B). Further evaluation of expression of TNFα signaling mediators in patients with differential *A20* levels demonstrated higher TNF receptor and *RelB* mRNA expression in tumors with elevated *A20* expression. These data suggest that A20 expression may be indicative of elevated TNFα or NF-κB signals. As the effects of these two pathways on GSC pro-tumorigenic behaviors are relatively unknown, more thorough evaluation of their biologies in the context of cancer stem cells may be warranted.

## Discussion

Our appreciation of the complex interactions between cell types during tumor initiation, progression, and recurrence continues to grow with our increasing understanding of cancer biology. Whereas researchers once considered tumors to be masses of clonal cancer cells, the involvement of immune cells, endothelial cells, and neighboring fibroblasts to tumor growth is now well recognized [1]. We believe that the identification of cancer stem cells with a probable concurrent stochastic clonality builds upon this model to recognize a previously underestimated contribution of the heterogeneity of tumor cells themselves [1]–[4]. By profiling the molecular and biological properties of cancer stem cells, we may therefore identify genes and proteins whose importance in cancer was poorly recognized. We have now determined that the inhibitor of apoptosis A20 is a cancer stem cell target. A20 was elevated in GSCs in comparison to non-stem glioma cells at both the mRNA and protein levels in cells isolated directly from glioma patient specimens and human glioma xenografts. Targeting *A20* expression with shRNA in GSCs significantly impaired their growth and survival in vitro and increased tumor latency in mice bearing human glioma xenografts. The importance of A20 to human glioma patients is further demonstrated by the association of elevated A20 levels with poor outcome.

Although current methods for cancer stem cell enrichment from solid cancers have been sufficient to differentiate tumor subpopulations, prospective identification of cancer stem cells has limitations that contribute to the controversy surrounding their existence [1]–[35]. Due to the restricted amount of tissue often available after pathologic review, it is difficult to generate enough patient-derived GSCs for the majority of experiments without culture or amplification as a xenograft. As we believe that microenvironmental conditions within the tumor contribute to GSC maintenance [74], we utilize a xenograft isolation system to obtain sufficient GSCs, but validated key studies with direct analysis of patient specimens. Experiments with cells directly derived from primary glioma specimens would be optimal, but results in GSCs from xenograft and patient-derived specimens have, thus far, been similar [11],[15]. Once tissue or xenografts are obtained, enrichment or depletion of cancer stem cells from the bulk tumor can be useful to respectively isolate glioma stem cell and non-stem glioma cell fractions with the stipulation that both GSC and non-stem glioma populations are heterogeneous and unlikely to be mutually exclusive based on current sorting protocols [1]–[4],[27]. Fluorescence-activated cell sorting (FACS) sorting to enrich for glioma stem cells has relied on the presence of the glycosylated form of the cell surface marker CD133 (Prominin-1): a protein whose role in tumorigenesis and glioma biology remains unclear [27]. However, CD133 is not the only marker useful for prospective identification of glioma stem cells, not all CD133+ cells are GSCs, and CD133 cannot exclusively segregate for tumorigenic potential and self-renewal in all glioma patient samples and cell lines studied [27]–[33]. The side population associated with ABCG2 transporter activity has been shown to be an important segregator for tumorigenic potential in mouse and human gliomas [24]–[33]. In gliomas without CD133 expression, the carbohydrate antigen SSEA-1/CD15/LeX can enrich for tumor-initiating cells [32]. CD133 and SSEA-1 separations are based on the use of antibodies against unspecified carbohydrate modified epitopes, adding further complexity by suggesting that posttranslational modifications to cell surface proteins are important for cancer stem cell biology [27]. These data suggest that, similar to leukemias, no one cell surface marker will be sufficient to isolate a homogeneous population of cancer stem cells from solid tumors. It is therefore enticing to suggest that molecular targets, such as A20, that are elevated in GSC-enriched fractions may segregate for a cancer stem cell subpopulation. Validating our findings in a model without dependence on CD133, we characterized A20 expression in lines with tumor enrichment in the SSEA-1+ fraction and found increased A20 expression (Figure S1). These results strongly suggest that A20 segregates with tumor initiating potential.

Our data with A20 expression suggest that, regardless of the cell surface markers used for isolation, the tumor-maintaining glioma subfractions may have common intracellular molecular targets. Determining the direct contribution of A20 (or cells expressing other identified nuclear targets such as HIF2α[11] and Bmi-1 [22]) is limited by our inability to sort for live A20-positive cells due to its intracellular localization. However, one recent study was able to utilize an indirect reporter based method to further elucidate the role of the transcription factor Oct4 in cancer stem cell biology. When the Oct4 promoter was used to drive green fluorescent protein (GFP) expression in osteosarcoma cells, reporter activity could identify tumor-initiating cells [75]. Application of similar methodologies to other cancer stem cell targets could permit the development of non-cell surface marker based sorting techniques and lead to further confirmation of the existence of cancer stem cells.

Although cancer stem cells can be isolated using different methodologies, a common theme in cancer stem cell biology is the ability of this tumor subpopulation to survive cellular assaults and repopulate the tumor. To date, only a few molecular mechanisms for GSC resistance to apoptotic signals have been identified. GSC radioresistance is linked to elevated checkpoint activation and DNA repair [15], whereas chemoresistance is associated with improved drug efflux due to the presence of the ABCG2 transporter [24]. Resistance to TRAIL-induced apoptosis in GSCs may be due to reduced levels of caspase 8 [19], and GSCs appear to be less sensitive to Fas-induced apoptosis due to decreased levels of oligomeric Fas [20]. Our data now add A20 as one of this growing list of prosurvival mediators in GSCs. We find that knockdown of *A20* induces apoptosis in GSCs and sensitizes GSCs to TNFα-induced apoptosis in cell culture, although we have not measured apoptosis specifically in the GSC compartment in vivo. Whether elevated levels of A20 in GSCs could also regulate other forms of therapeutic resistance remains to be investigated, but it is interesting to note that *A20* was one of a set of genes identified as mediators of resistance to O6-alkylating agents [52]. Cell lines were derived from primary and recurrent tumors selected for resistance to 1,3-bis (2-chloroethyl)-1-nitrosourea (BCNU) or temozolomide in media with 20% fetal calf serum [52]. Under this prodifferentiating condition, down-regulation of A20 and the established pluripotency gene leukemia inhibitory factor (LIF) were both associated with chemoresistance [52]. Roles for A20 (our data) and LIF [34] in GSC self-renewal imply that elevation of these proteins would be more likely to facilitate rather than impede GSC mediated chemoresistance. These data reinforce the notion that the differentiation state of the glioma cells may differentially impact the mechanisms through which tumor cells survive cellular stresses (i.e., conditional essentiality).

Our examination of A20 in the context of glioma heterogeneity revealed A20 contributes to GSC protumorigenic behaviors, but recent evidence in the literature suggests a tumor suppressive role for A20 in other cancers. Although we find A20 is highly expressed in GSCs and point mutations in A20 have not been identified in glioma [72],[73], *A20* deletion and mutation is prevalent in lymphoma [46]–[51]. *A20* knockdown in GSCs promotes apoptosis and reduces tumorigenic potential in mouse models of human cancer, but overexpression of A20 in A20-deficient lymphoma cells produces similar results [46],[47]. Together, these data suggest that A20 may be a tumor suppressor or a tumor enhancer depending on the cancer type. Many molecular pathways, including the NF-κB pathway which A20 can inhibit, may be either pro- or antitumorigenic depending on the cellular context and tumor stage [59]. For example, inhibition of NF-κB signals in mouse epidermis resulted in squamous cell carcinomas [76], whereas a similar transgenic strategy in transformed hepatocytes prevented tumor progression to hepatocellular carcinoma [77]. A20 may similarly have differential effects on tumor development or progression depending on the biological requirements for A20 in specific tissues. Indeed, unique roles for A20 in lymphoma and glioma tumor biology may be anticipated based on differences in basal expression between lymphoid tissues and the brain. In the majority of tissues, including brain, A20 expression is induced by a variety of stimuli (TNFα, lipopolysaccharide, interleukin-1), but unexpressed or expressed at very low levels under basal conditions [53]. However, A20 is constitutively expressed in lymphoid tissues, particularly the thymus and lymph nodes where A20 is critical for suppression of inflammatory responses mediated by NF-κB [53],[57]. Mutation of *A20* may therefore be more beneficial during the development of lymphoma. In contrast, our analysis suggests A20 levels increase with brain tumor grade, suggesting a benefit for A20 elevation in astrocytoma growth and linking A20 to glioma tumor progression. Thus, the precise biological and molecular outcomes of targeting *A20* in each tumor type must be further defined, particularly before broadly applying A20-based therapies for cancer treatment.

Our data implicate A20 as an important mediator of cancer stem cell biology by demonstrating that A20 is involved in glioma maintenance through the regulation of GSC growth and survival. The increased survival of mice bearing intracranial tumors upon *A20* targeting, and the decreased survival of glioma patients with elevated mRNA, both indicate that inhibition of A20 (or its downstream mediators) may be beneficial for glioma therapy. However, the increased survival of mice upon A20 restoration to A20-deficient lymphoma cells demonstrates that targeting A20 may be harmful for other tumor types. As we do not fully understand the mechanisms that cause A20 to have differential effects on tumor growth and cancer cell behaviors, further elucidation of A20 molecular and biological signals is warranted.

## Materials and Methods

### Ethics Statement

Primary human brain tumor patient specimens were obtained from patients providing informed consent under protocols approved by the Duke University or Cleveland Clinic Institutional Review Boards. All animal experiments were performed in accordance with a Duke University or Cleveland Clinic Institutional Animal Care and Use Committee–approved protocol.

### Isolation and Culture of Matched GSCs and Non-Stem Glioma Cells

As previously described [11],[12],[15],[21],[23],[25], matched cultures enriched or depleted for GSCs were isolated from primary human brain tumor patient specimens directly or those passaged short term in immunocompromised mice. A Papain Dissociation System (Worthington Biochemical) was used to dissociate tumors according to the manufacturer's instructions (detailed protocol: http://www.worthington-biochem.com/PDS/default.html). Cells were then cultured in Neurobasal medium supplemented with B27 without vitamin A, l-glutamine, sodium pyruvate (Invitrogen), 10 ng/ml basic fibroblast growth factor (bFGF), and 10 ng/ml epidermal growth factor (EGF) (R&D Systems) for at least 6 h to recover surface antigens. Cells were then labeled with an allophycocyanin (APC)-conjugated CD133 antibody (Miltenyi Biotec), and sorted by fluorescence-activated cell sorting (FACS). Alternatively, cells were separated microbead-conjugated CD133 antibodies and magnetic columns (Miltenyi Biotec). CD133-positive cells were designated as GSCs whereas CD133-negative cells were designated as non-stem glioma cells. Consistent with previously defined methods for GSC and non-GSC cell culture [10], GSCs were cultured in the earlier-defined medium: matched non-stem glioma cells were cultured for at least 24 h in 10% serum containing DMEM to allow cell survival. After recovery, DMEM medium was removed and the cells cultured in supplemented Neurobasal medium for at least 12 h before experiments were performed in identical medium. The cancer stem cell nature of the CD133-positive cells was confirmed by fluorescent in situ hybridization (FISH) analysis, serial neurosphere assays, and tumor formation assays, but cultures depleted of cancer stem cells did not self-renew and or initiate tumors (unpublished data).

### Real-Time PCR

Total RNA was prepared using the RNeasy kit (Qiagen), and reverse transcribed into cDNA using a SuperScript III First-Strand Synthesis Kit (Invitrogen). To investigate expression of *A20* and *Olig2*, individual gene primers were ordered from Integrated DNA technologies and Master Mixes were purchased from SuperArray Bioscience Corporation. mRNA levels were measured using an ABI-7900 system (Applied Biosystems). Sequences for primer sets were as follows:

*A20*: Forward 5′-AGT GTT CCC AGG TGG CCT TAG AAA-3′; Reverse 5′-TCT CAG CCA AGA CGA TGA AGC AGT-3′. *Olig2*: Forward 5′-GGT AAG TGC GCA ATG GTA AGC TGT-3′; Reverse 5′-TAC AAA GCC CAG TTT GCA ACG CAG-3′.

### Immunofluorescence Staining

Cells, neurospheres, or tumor sections were fixed with 4% paraformaldehyde, washed with Tris-buffered saline, and incubated with polyclonal mouse anti-A20 (Santa Cruz Biotechnology) and goat anti-Sox2 (Santa Cruz Biotechnology) where indicated. Primary antibodies were incubated for 16 h at 4°C followed by detection with Alexa 488 donkey anti-mouse (Invitrogen) and Alexa 568 donkey anti-rabbit (Invitrogen) secondary antibodies. Nuclei were stained with Hoechst 33342 (Invitrogen), and slides were mounted using Fluoromount (Calbiochem). Images were taken with a Leica SP-5 confocal microscope.

### Western Blotting and Antibodies

Equal amounts of cell lysate were resolved by SDS-PAGE, transferred to polyvinylidene difluoride membranes (Millipore), and detected using an enhanced chemiluminescence system (Pierce Biotechnology) with antibodies against A20 (Abcam or Santa Cruz Biotechnology), Olig2 (R&D Systems), and Tubulin (Sigma).

### FACS Analysis for CD133 and A20 Costaining

Tumors were dissociated as described earlier and bulk or isolated GSCs, and non-stem glioma cells were fixed in 4% paraformaldehyde and subjected to FACS analysis. FACS analysis was performed on a FACS Aria with 100-µm nozzle and low sheath pressure. Human-specific anti-CD133 (293C3) conjugated to allophycocyanin5 (APC) (Miltenyi) was used with anti–A20-PE generated using the Lightning-Link PE kit (Innova Biosciences) in combination with an A20 antibody (Abcam).

### Lentiviral-Mediated shRNA Targeting

Lentiviral shRNA clones (Sigma Mission RNAi) targeting *A20* and a scrambled nontargeting control (SHC002) were purchased from Sigma. These vectors were cotransfected with the packaging vectors psPAX2 and pCI-VSVG (Addgene) or the ViraPower Lentiviral Expression System packaging mix (Invitrogen) into 293FT cells by Lipofectamine 2000 (Invitrogen) to produce the virus. Efficiency of different lentiviral shRNA clones in cells was determined by Western blot analysis and real-time PCR. The sequence of the shRNAs utilized for shRNA1 (NM_006290.2-635s1c1), shRNA2 (NM_006290.2-2104s1c1), and shNRA3 (NM_006290.2-957s21c1) was as follows: 5′-CCGGCACTGGAAGAAATACACATATCTCGAGATATGTGTATTTCTTCCAGTGTTTTTG-3′ and 5′-CCGGGAAGCTCAGAATCAGAGATTTCTCGAGAAATCTCTGATTCTGAGCTTCTTTTTG-3′; and 5′-GTACCGGGATGAAGGAGAAGCTCTTAAACTCGAGTTTAAGAGCTTCTCCTTCATCTTTTTTG-3′.

### Cell Viability Assay

GSCs infected with lentivirus expressing the indicated shRNAs for 24 h were plated in 96-well plates at 1,000 cells per well. Cell titers were determined after the indicated number of days after plating using the CellTiter-Glo Luminescent Cell Viability Assay kit (Promega).

### Cell-Cycle Analysis and Annexin V Staining

GSCs plated in six-well plates at 100,000 cells per well were infected with lentivirus expressing the indicated shRNAs for 48 hours. To determine the percentage of cells in each phase of the cell cycle, cells were fixed with ethanol and stained with propidium iodide followed by cell-cycle analysis. To detect apoptotic cells, Annexin V-FITC staining was performed with the Annexin V-FITC Apoptosis Detection Kit (BD Pharmingen) according to the manufacturer's instructions. For experiments in which GSCs and matched non-stem glioma cells were treated with TNFα, cells were plated at a density of 100,000 cells per well in a six-well plate and treated for 72 h with 5 ng/ml TNFα.

### Caspase 3/7 Assay

GSCs infected with lentivirus expressing the indicated shRNAs for 24 h were plated at 1,000 cells per well and caspase 3/7 activity measured with a commercially available kit (Promega) after an additional 24 h. Relative caspase activity was then determined by correcting for cell titers determined as indicated above. When TNFα treatment was combined with shRNA treatments, cells were infected with lentivirus expressing the indicated shRNAs for 24 h followed by TNFα treatment for 24 h.

### TUNEL Staining

GSCs infected with lentivirus expressing the indicated shRNAs for 36 h were stained for TUNEL using an Apo-BrdU-Red In Situ DNA Fragmentation Assay Kit (Biovision) according to the manufacturer's instructions.

### Neurosphere Formation Assay and Quantification of Neurosphere Volume

GSCs infected with lentivirus expressing the indicated shRNAs for 24 h were plated in 24-well plates at 10 cells per well and the percentage of wells containing neurospheres quantified at indicated times. For secondary sphere formation, neurosphere forming cells from the first plating were trypsinized and plated at 10 cells per well in 24-well plates. Neurospheres were imaged with an Olympus CK40 digital camera mounted to a light microscope and neurosphere size was calculated using ImageJ software. When TNFα treatment was combined with shRNA treatments, cells were infected with lentivirus expressing the indicated shRNAs for 24 h followed by treatment with 5 ng/ml TNFα treatment.

### Intracranial Tumor Assays

Intracranial transplantation of GSCs into nude mice was performed as described [11],[12],[15],[23],[25] in accordance with a Duke University or Cleveland Clinic Institutional Animal Care and Use Committee approved protocol. Briefly, 36 h after lentiviral infection, cells were counted and the indicated number of live cells implanted into the right frontal lobes of athymic nude mice. Mice were maintained until the development of neurological signs.

### Statistical Analysis

Significance was tested by *t*-test or ANOVA using GraphPad InStat 3.0 software. For repeated measures ANOVA and in vivo studies where Kaplan-Meier curves and log-rank analysis were performed, MedCalc software was used.

## Supporting Information

Figure S1 **A20 is elevated in SSEA-1+ fractions of human glioma cells where SSEA-1, but not CD133, is informative for tumorigenic potential.** Equal amounts of lysates from the human glioma cell lines 1228 and 905 sorted for the expression of SSEA-1 were probed for the expression of A20 by Western blot. α-Tubulin was utilized as a loading control.
(0.06 MB TIF)

Figure S2 **Flow cytometry demonstrates A20 colocalizes with a glioma stem cell marker.** (A and B) Flow cytometry of fixed bulk tumor cells isolated directly from T3832 (A) or T3946 (B) patient specimens demonstrates significant co-staining of the glioma stem cell marker CD133 and A20. (C and D) Flow cytometry analysis of glioma stem cell-enriched (C) and -depleted (D) cultures from a T4105 patient specimen passaged short term in immunocompromised mice demonstrates passage of cells in vitro maintains coexpression of the glioma stem cell marker CD133 and A20 in glioma stem cells with reduced CD133 and A20 expression in non-stem glioma cells.
(1.83 MB TIF)

Figure S3 **A20 preferentially decreases the growth of glioma stem cells.** Cell growth as measured with Trypan Blue staining demonstrated that *A20* shRNA decreases the growth of non-stem glioma cells isolated from T4121 (A) or T08-836 cells (B). An asterisk (*) indicates *p*<0.01 with ANOVA comparison to non-targeting shRNA. Matched glioma stem cells with *A20* targeting are shown in Figure 3C and 3D. When the fold change in cell numbers relative to the average non-targeting shRNA cell number is calculated for T4121 (C and D) or T08-837 (E and F) cells, targeting with *A20* shRNA1 (C and E) or *A20* shRNA3 (D and F) demonstrates significantly greater reductions in cell number in the glioma stem cell fractions. An asterisk (*) indicates *p*<0.001 with *t*-test comparison to non-stem glioma cells.
(0.47 MB TIF)

Figure S4 **A20 protects GSCs from TNFα-induced apoptosis.** GSC enriched cultures isolated from a T4597 glioma xenograft were infected with non-targeting shRNA or shRNA directed against *A20* and treated with 5 ng/ml TNFα. (A) Relative caspase activity increased with *A20* knockdown and was further increased by TNFα treatment. (B) Neurosphere formation decreased with *A20* knockdown and was further decreased by TNFα treatment. An asterisk (*) indicates *p*<0.05 with ANOVA comparison to similarly treated nontargeting control cells. A number sign (#) indicates *p*<0.05 with ANOVA comparison of TNF to untreated cells infected with the same shRNA.
(0.13 MB TIF)

Figure S5 **Tumors resulting from implantation of** ***A20*** **knockdown GSCs express A20.** Immunofluorescence of paraffin-embedded sections of tumors resulting from implantation of GSCs infected with nontargeting shRNA or *A20* shRNA1 demonstrates A20 is expressed in both tumor types.
(0.41 MB TIF)

Figure S6 **Glioblastoma genetic subsets and expression of A20.** (A) Table demonstrating the number of samples in the TCGA database analyzed as having low, intermediate, or high A20 expression. The number of samples in each group indicated as having mutations in p53, EGFR, PTEN, or NF1 is also shown. (B) Analysis of the percentage of patients with mutations in p53, EGFR, PTEN, or NF1 for the groups have differential A20 expression is shown. The percentage of patients with p53 or NF1 mutations is increased in the set of patients with high A20 expression. (C) Some TNFα signaling mediator mRNAs are elevated in the set of patients with high A20 expression. Analysis of the expression of multiple TNF receptor and NF-κB family members demonstrated elevated expression was often observed in patient samples with elevated A20.
(0.51 MB TIF)

## Abbreviations

FACS: fluorescence-activated cell sorting
GSC: glioblastoma stem cell
shRNA: short hairpin RNA
