# Supplementary figures and images for: Targeting A20 Decreases Glioma Stem Cell Survival and Tumor Growth
Source: PLoS Biol. 2010 Feb 23;8(2):e1000319. doi: 10.1371/journal.pbio.1000319 (PMC2826371; doi:10.1371/journal.pbio.1000319)

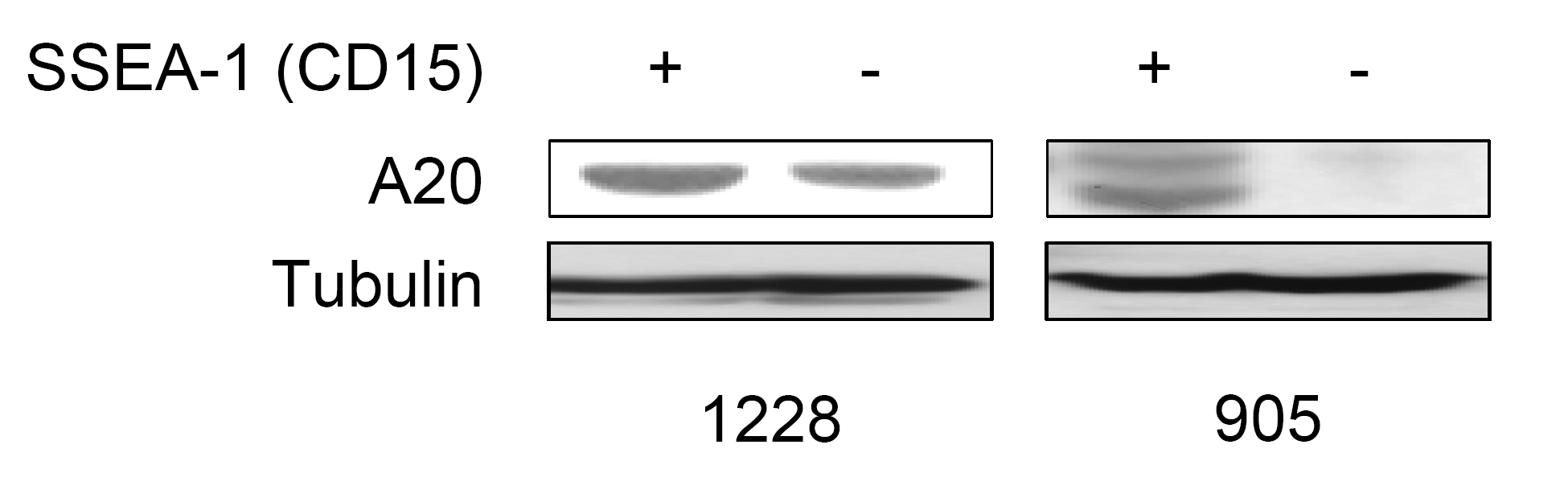

Supplement: Figure S1 — A20 is elevated in SSEA-1+ fractions of human glioma cells where SSEA-1, but not CD133, is informative for tumorigenic potential. Equal amounts of lysates from the human glioma cell lines 1228 and 905 sorted for the expression of SSEA-1 were probed for the expression of A20 by Western blot. α-Tubulin was utilized as a loading control. (0.06 MB TIF) [file pbio.1000319.s001.tif]

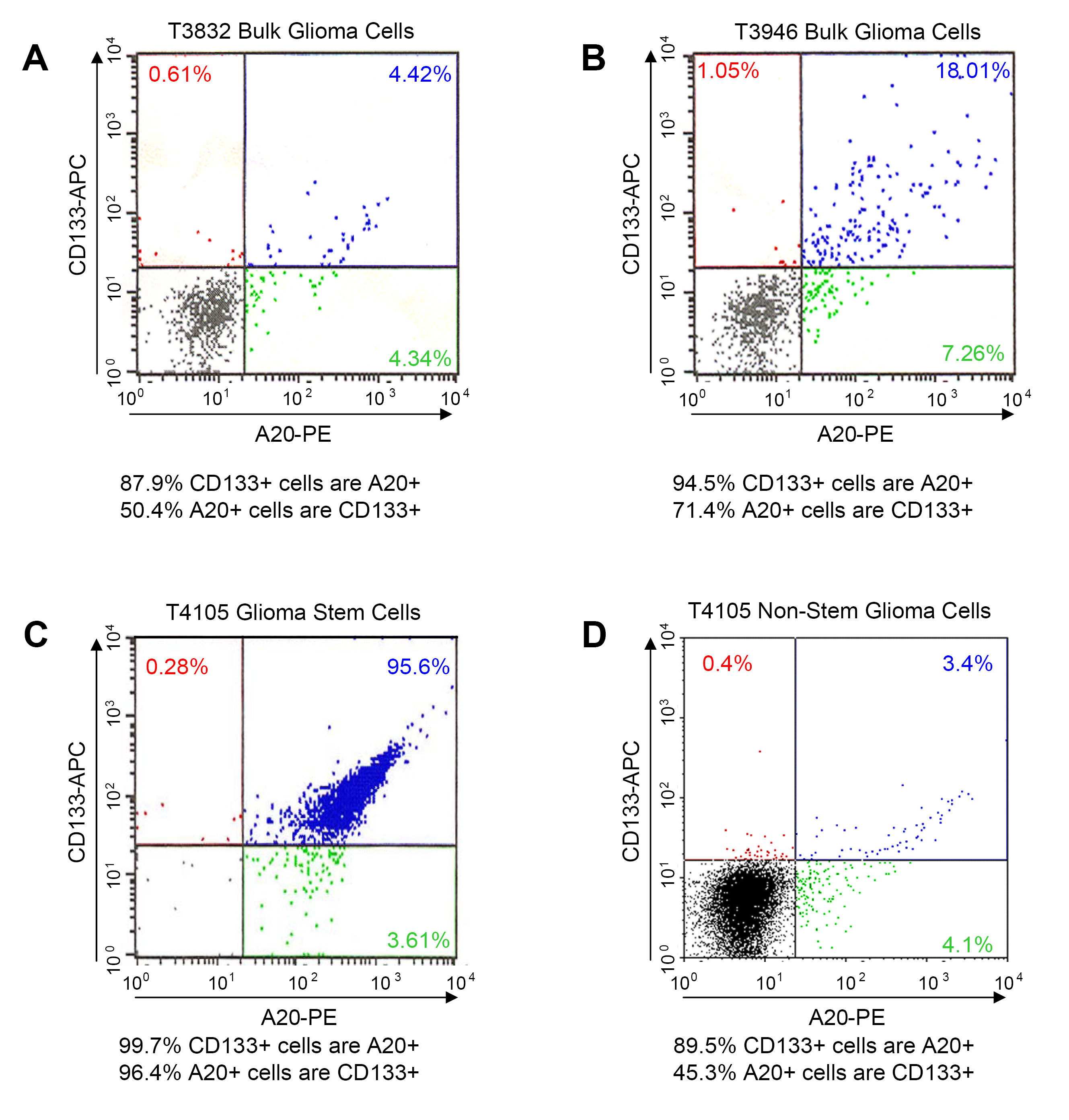

Supplement: Figure S2 — Flow cytometry demonstrates A20 colocalizes with a glioma stem cell marker. (A and B) Flow cytometry of fixed bulk tumor cells isolated directly from T3832 (A) or T3946 (B) patient specimens demonstrates significant co-staining of the glioma stem cell marker CD133 and A20. (C and D) Flow cytometry analysis of glioma stem cell-enriched (C) and -depleted (D) cultures from a T4105 patient specimen passaged short term in immunocompromised mice demonstrates passage of cells in vitro maintains coexpression of the glioma stem cell marker CD133 and A20 in glioma stem cells with reduced CD133 and A20 expression in non-stem glioma cells. (1.83 MB TIF) [file pbio.1000319.s002.tif]

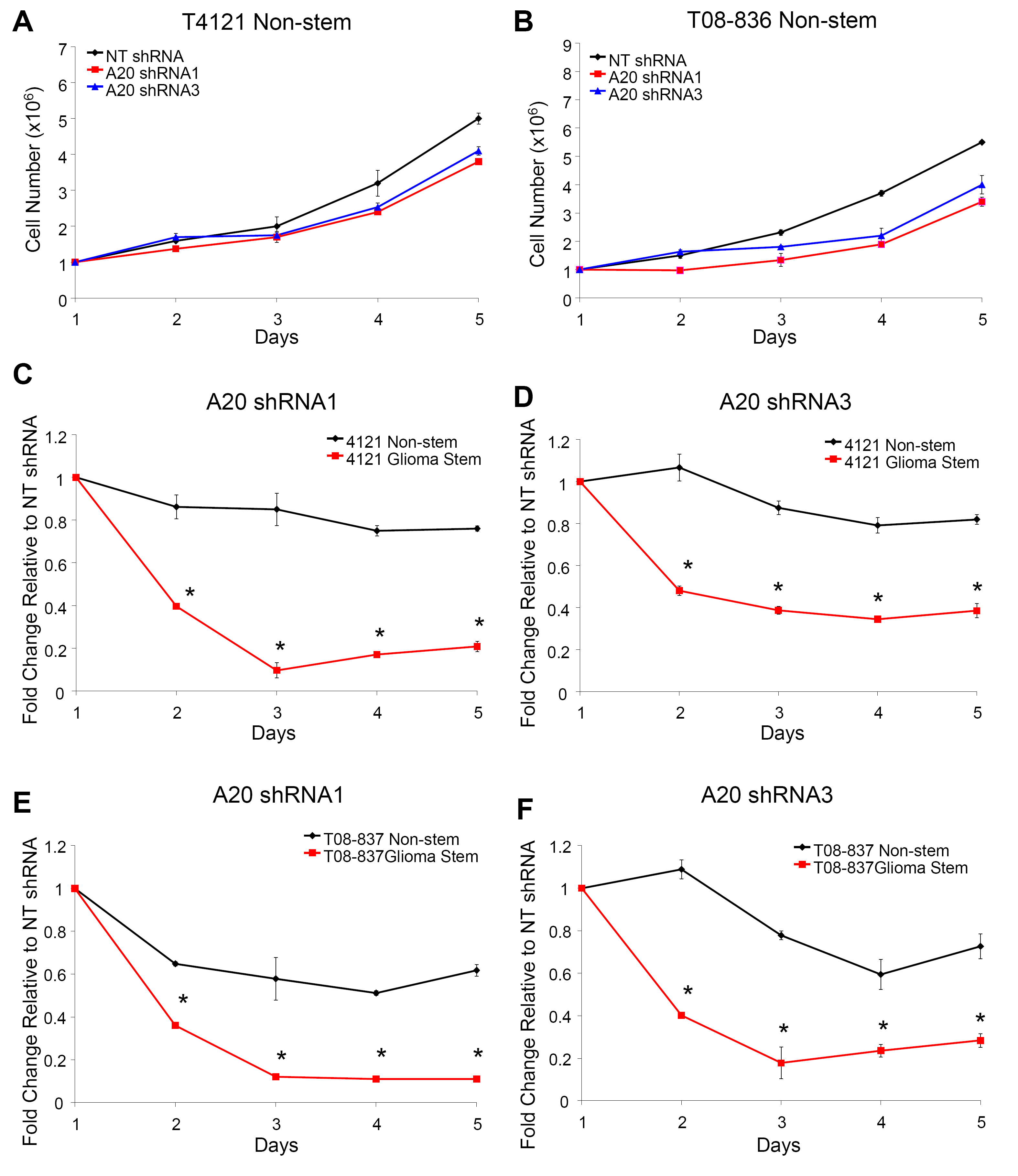

Supplement: Figure S3 — A20 preferentially decreases the growth of glioma stem cells. Cell growth as measured with Trypan Blue staining demonstrated that A20 shRNA decreases the growth of non-stem glioma cells isolated from T4121 (A) or T08-836 cells (B). An asterisk (*) indicates p<0.01 with ANOVA comparison to non-targeting shRNA. Matched glioma stem cells with A20 targeting are shown in Figure 3C and 3D. When the fold change in cell numbers relative to the average non-targeting shRNA cell number is calculated for T4121 (C and D) or T08-837 (E and F) cells, targeting with A20 shRNA1 (C and E) or A20 shRNA3 (D and F) demonstrates significantly greater reductions in cell number in the glioma stem cell fractions. An asterisk (*) indicates p<0.001 with t-test comparison to non-stem glioma cells. (0.47 MB TIF) [file pbio.1000319.s003.tif]

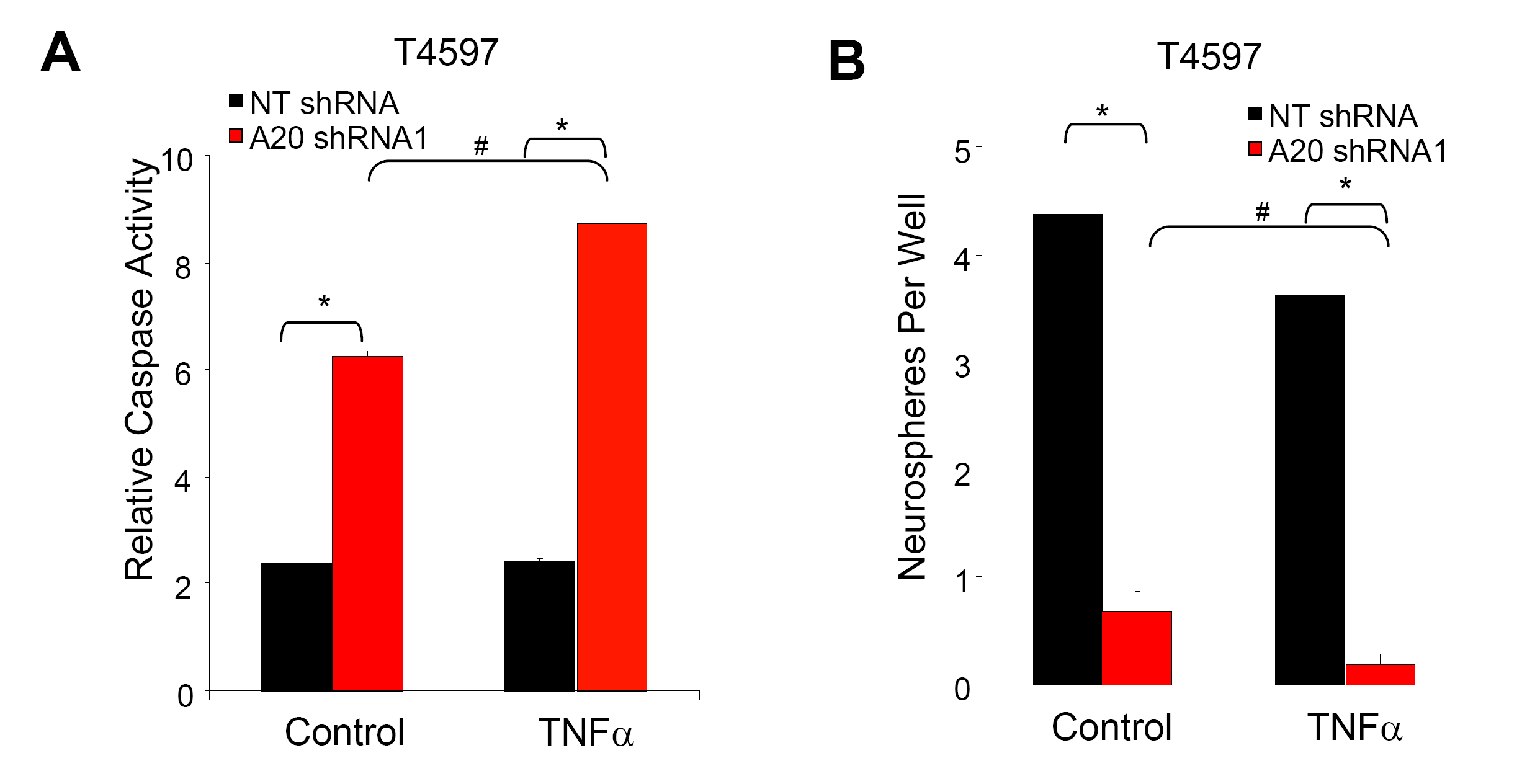

Supplement: Figure S4 — A20 protects GSCs from TNFα-induced apoptosis. GSC enriched cultures isolated from a T4597 glioma xenograft were infected with non-targeting shRNA or shRNA directed against A20 and treated with 5 ng/ml TNFα. (A) Relative caspase activity increased with A20 knockdown and was further increased by TNFα treatment. (B) Neurosphere formation decreased with A20 knockdown and was further decreased by TNFα treatment. An asterisk (*) indicates p<0.05 with ANOVA comparison to similarly treated nontargeting control cells. A number sign (#) indicates p<0.05 with ANOVA comparison of TNF to untreated cells infected with the same shRNA. (0.13 MB TIF) [file pbio.1000319.s004.tif]

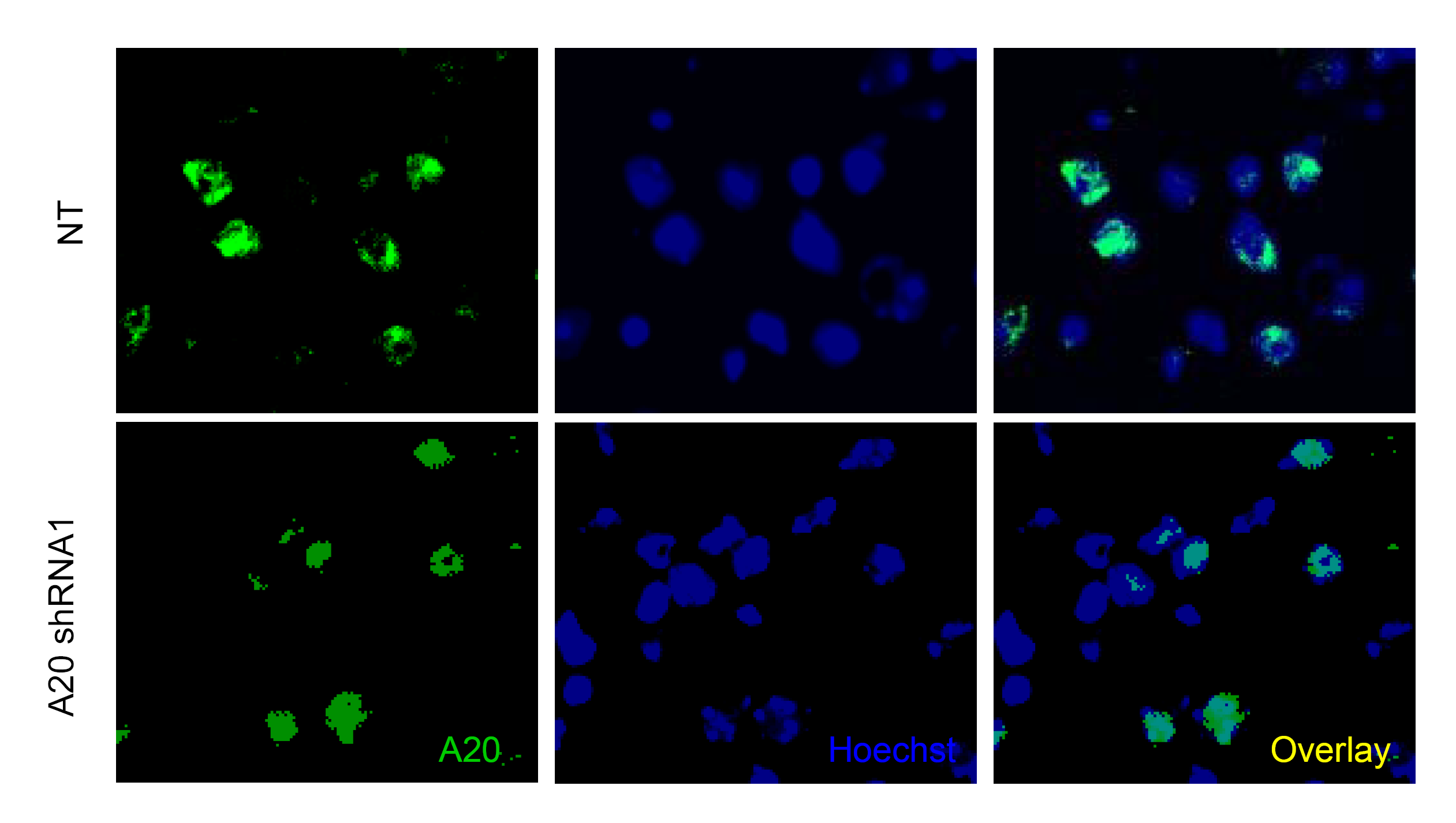

Supplement: Figure S5 — Tumors resulting from implantation of A20 knockdown GSCs express A20. Immunofluorescence of paraffin-embedded sections of tumors resulting from implantation of GSCs infected with nontargeting shRNA or A20 shRNA1 demonstrates A20 is expressed in both tumor types. (0.41 MB TIF) [file pbio.1000319.s005.tif]

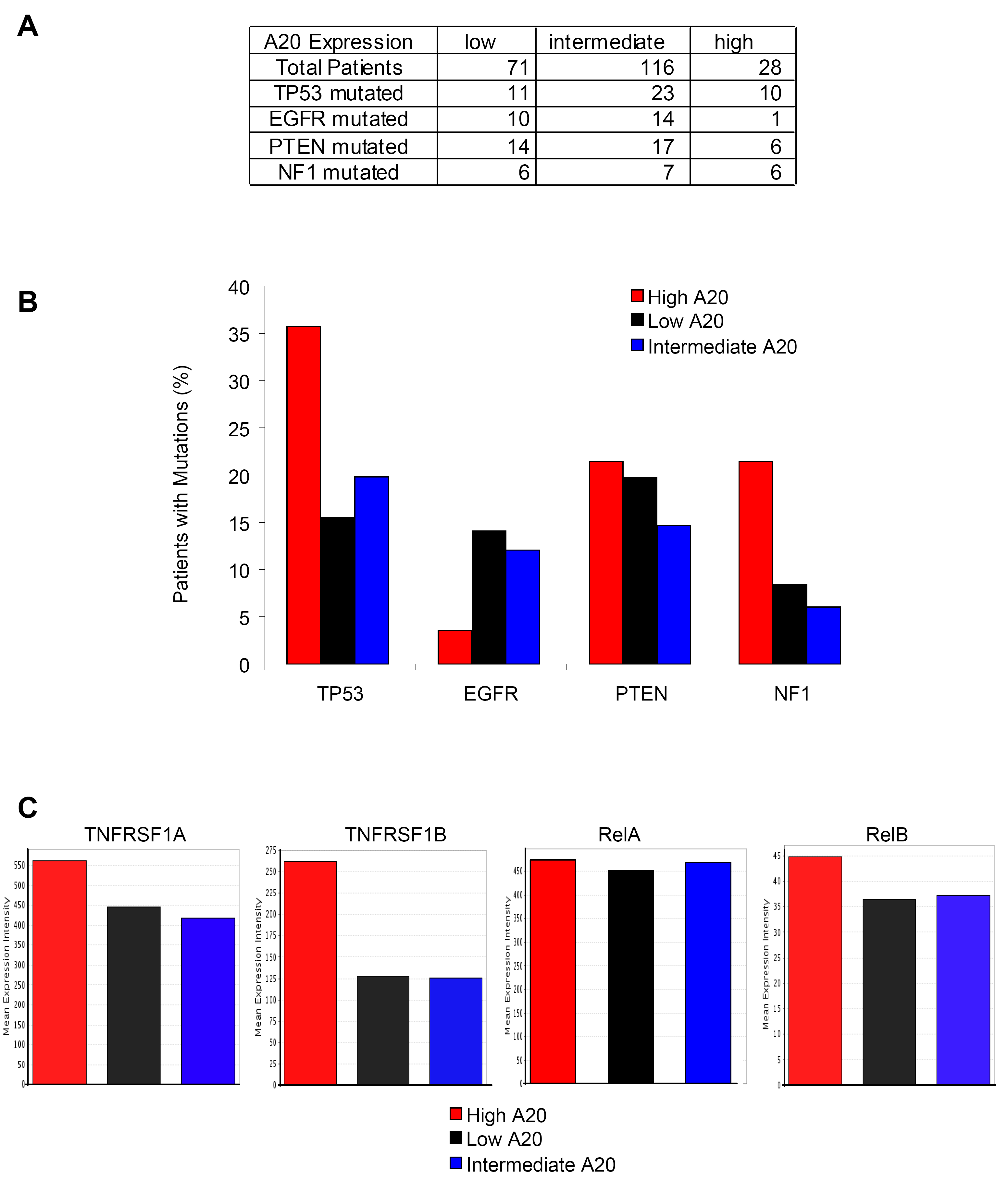

Supplement: Figure S6 — Glioblastoma genetic subsets and expression of A20. (A) Table demonstrating the number of samples in the TCGA database analyzed as having low, intermediate, or high A20 expression. The number of samples in each group indicated as having mutations in p53, EGFR, PTEN, or NF1 is also shown. (B) Analysis of the percentage of patients with mutations in p53, EGFR, PTEN, or NF1 for the groups have differential A20 expression is shown. The percentage of patients with p53 or NF1 mutations is increased in the set of patients with high A20 expression. (C) Some TNFα signaling mediator mRNAs are elevated in the set of patients with high A20 expression. Analysis of the expression of multiple TNF receptor and NF-κB family members demonstrated elevated expression was often observed in patient samples with elevated A20. (0.51 MB TIF) [file pbio.1000319.s006.tif]
